# Supplementary material for: Predation and fragmentation portrayed in the statistical structure of prey time series
Source: BMC Ecol. 2009 May 6;9:10. doi: 10.1186/1472-6785-9-10 (PMC2689204; doi:10.1186/1472-6785-9-10)
Supplement: Additional file 2 — Voles and related classes ODDox Documentation. ODDox documentation of the agent-based model (ALMaSS) applied by Hendrichsen et al. The documentation is started by activating main.html. [file 1472-6785-9-10-S2.zip › Vole_ODDox/globals_type.html]

ALMaSS ODDox: Class Members

- Main Page
- Related Pages
- Classes
- Files

- File List
- File Members

- All
- Functions
- Variables
- Typedefs
- Enumerations
- Enumerator
- Defines

- AnsiString
  : PopulationManager.h- base\_generator\_type
    : BoostRandomGenerators.h- distribution\_type
      : BoostRandomGenerators.h- gen\_type\_int
        : BoostRandomGenerators.h- TListOfAnimals
          : PopulationManager.h- TListOfPredators
            : Predators.H- uint32
              : GeneticMaterial.H- Vole\_FemaleList
                : VolePopulationManager.H- Vole\_MaleList
                  : VolePopulationManager.H

---

Generated on Thu Jan 22 14:13:48 2009 for ALMaSS ODDox by 
 1.5.6
